# A Study on the Effect of Metabolic Heat Generation on Biological Tissue Freezing

**DOI:** 10.1155/2013/398386

**Published:** 2013-11-05

**Authors:** Sonalika Singh, Sushil Kumar

**Affiliations:** Department of Applied Mathematics and Humanities, S. V. National Institute of Technology, Surat, Gujarat 395007, India

## Abstract

The effect of metabolic heat generation on the freezing of biological tissue has been studied. Quasi-steady approximation is used to solve the Pennes bioheat equation in tissues. Temperature profile and motion of freezing interfaces are obtained for different values of metabolic heat generation. It is observed that metabolism has a significant effect on freezing of biological tissues during cryosurgery.

## 1. Introduction

The effect of volumetric energy generation on phase change problems is important in several applications including nuclear energy, geophysics, material processing, vivo freezing of biological tissues, and solar collectors. Cryosurgery is one of the examples that involves freezing of biological tissue in vivo. In tissues, heat is generated by metabolism and blood perfusion, and the heat, that is, generated during metabolic processes such as growth and energy production of the living system, is defined as metabolic heat. 

Cryosurgery is one of the most important therapies for tumor treatment. In cryosurgery, extreme cold is used to destroy the tissue for therapeutic purpose. It involves local freezing of tissues for their controlled destruction or removal. In 1960, the concept of injecting liquid nitrogen through cryoprobe into the target tissue to freeze them from within was introduced. Several advantages of cryosurgery include the low invasiveness of the procedure, minimal blood flow, localizing the site of surgery, and reducing the recovery and hospitalization time for the patient. The earliest model of heat transfer in the biological tissue is discussed by Pennes [1]. Cryosurgery destroys cells and tissues by a complex mechanism containing ice-related factors [2]. Advantages of cryosurgery have initiated interest among researchers to apply it to the field of skin, breast, prostate, liver, and lung cancers [3–11].

The aim of cryosurgery is to maximize the damage to the undesired tissues within the defined domain and minimize the injury to the surrounding healthy tissues [9, 12]. The parameters which influence the process of cryosurgery are the coolest temperature in the tissue, the duration of frozen cycle, the rate of freezing front propagation, the thawing rate, and the freezing-thawing cycles [13–21]. The factors which affect necrosis such as the lowest temperature in the tissue or the rate of freezing front propagation depend on the biophysical parameters that are present in a given cryosurgical procedure, some of which may be selected and controlled by the surgeons. These parameters include the temperature and duration of freezing-thawing process, the shape and size of cryoprobe, the heat capacity and thermal conductivity of the tissue, the rate of blood flow, and metabolism in the involved tissue [22]. Gage et al. [16] have studied the effect of varying freezing rate, duration of freezing and thawing cycles to investigate the effect of these factors on cell destruction in dog skin. They suggested that features like fast cooling, slow thawing, and repetition of the freeze/thaw cycle should be modified by maintaining the tissues in the frozen state for several minutes and slow thawing.

 Blood perfusion and metabolic heat generation also have an important effect on heat transfer in tissues [23–26]. The coolest temperature in the tissues is one of the crucial points in the process of cryosurgery. Moreover, the duration of frozen state also has much influence on the success of cryosurgery [16, 27–31]. The tissue destruction is increased when it is held in the frozen state in the temperature range at which recrystallization occurs [14]. A common problem in cryosurgery is the extent of post operative bleeding caused by parenchyma fractures and related to the thermal stress inside the target tissue [32]. Shi et al. [33] have described the large volumetric expansion having the primary contributor to large stress development during the freezing of biomaterial through ice-crystallization. The thermal gradient and the effect of volumetric expansion associated with freezing are the two most important factors that induce thermal stress [33, 34]. Consequently, the study of the thermal gradient inside the tissue is also an important issue for the optimization of cryosurgery.

The temperature transients in tumour and normal tissue are useful to say whether the tumour is damaged or not and to minimize the injury to healthy tissues during cryosurgery. There is a need for a simple analytical solution to evaluate the effect of parameters like metabolic heat generation on ice-crystallization. A process of simplification is used in solving a variety of problems which can eliminate the need for numerical solutions. In this paper, the method of quasi-steady approximation to study the effect of metabolic heat generation on one-dimensional ice-crystallization during cryosurgery has been used. Temperature profiles and motion of freezing interface are obtained for different values of metabolic heat generation.

## 2. Mathematical Model

**Figure 1:**
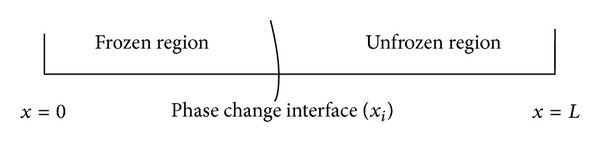
Schematic representation of one-dimensional model.

In the present study, one-dimensional ice-crystallization in biological tissue of length *L* has been considered as shown in Figure 1. Cryoprobe with temperature *T*_0_ = −196°C is applied at *x* = 0, while at the other end *x* = *L* an adiabatic condition is used. In the frozen region, blood perfusion and metabolic heat generation are zero [4, 5, 10, 11, 13].

The governing equations for one-dimensional ice-crystallization in biological tissue are as follows.

*In frozen region:*

(1) $$\begin{array}{c} \rho_{f}c_{f}\frac{\partial T_{f}}{\partial t}=k_{f}\frac{\partial^{2}T_{f}}{\partial x^{2}}\;\text{for}\;\;0\leq x\leq x_{i}. \end{array}$$

*In unfrozen region:*

(2) $$\begin{array}{c} \rho_{u}c_{u}\frac{\partial T_{u}}{\partial t}=k_{u}\frac{\partial^{2}T_{u}}{\partial x^{2}}+q_{b}+q_{m}\;\text{for}\;\;x_{i}\leq x\leq L. \end{array}$$

*Initial conditions:*

(3) Tu(x,0)=TI=37C°,Tf(x,0)=TI=37C°.

*Boundary conditions:*

(4) Tf(0,t)=T0=−196C°,∂Tu(L,t)∂x=0.

*Conditions at phase change interface:*

(5) $$\begin{array}{c} T_{f}\left(x_{i},t\right)=T_{\text{ph}}=T_{u}\left(x_{i},t\right),\\ k_{f}\frac{\partial T_{f}\left(x_{i},t\right)}{\partial x}-k_{u}\frac{\partial T_{u}\left(x_{i},t\right)}{\partial x}=\rho_{u}l\frac{dx_{i}}{dt}, \end{array}$$

where *ρ* is the density of tissue; *c* the specific heat; *k* the thermal conductivity; *x*_*i*_ the interface position of freezing front; *T* the temperature; *x* the space coordinate; *t* the time; *q*_*b*_ the blood perfusion term; *l* the latent heat of fusion; and *q*_*m*_ the metabolic heat generation in the tissue. Subscripts *u* and *f* are for unfrozen and frozen state, respectively, and ph and *I* are for phase change and initial states, respectively. Assuming the negligible effect of blood perfusion and using the following dimensionless variables and constants:

(6) $$\begin{array}{c} \alpha_{f}=\frac{k_{f}}{\rho_{f}c_{f}},\;\;\alpha_{u}=\frac{k_{u}}{\rho_{u}c_{u}},\;\;\alpha^{*}=\frac{\alpha_{u}}{\alpha_{f}},\\ x^{*}=\frac{x}{L},\;\;t^{*}=\frac{\alpha_{u}t}{L^{2}}\text{Ste},\;\;K^{*}=\frac{k_{u}}{k_{f}},\\ T_{f}^{*}=\frac{T_{f}-T_{0}}{T_{\text{ph}}-T_{0}},\;\;T_{u}^{*}=\frac{T_{u}-T_{0}}{T_{\text{ph}}-T_{0}},\\ q_{m}^{*}=\frac{q_{m}L^{2}}{k_{u}\left(T_{\text{ph}}-T_{0}\right)}, \end{array}$$

where Ste is the Stefan number defined as Ste = *c*_*u*_(*T*_ph_ − *T*_0_)/*l*.

Equations (1) and (2) become

(7) $$\begin{array}{c} \text{Ste}\;\alpha^{*}\frac{\partial T_{f}^{*}}{\partial t^{*}}=\frac{\partial^{2}T_{f}^{*}}{\partial x^{*2}}\;\text{for}\;\;0\leq x^{*}\leq x_{i}^{*},\\ \text{Ste}\;\frac{\partial T_{u}^{*}}{\partial t^{*}}=\frac{\partial^{2}T_{u}^{*}}{\partial x^{*2}}+q_{m}^{*}\;\text{for}\;\;x_{i}^{*}\leq x^{*}\leq 1. \end{array}$$

The initial and boundary conditions (3)-(4) become

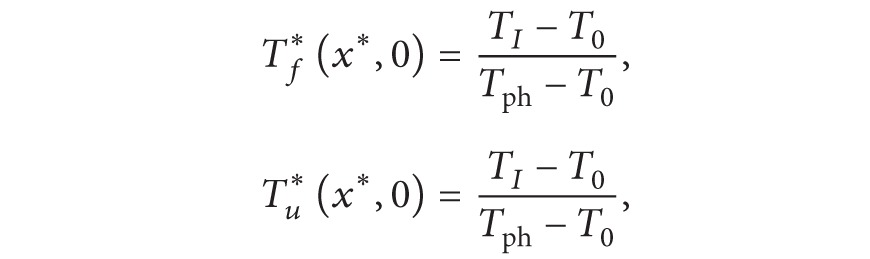
(8)

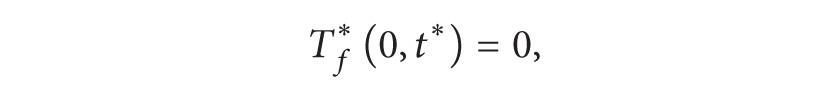
(9)

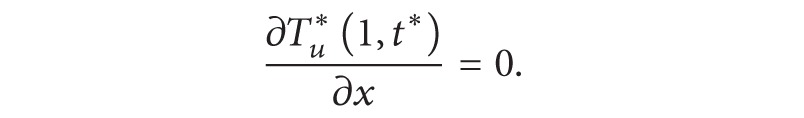
(10)
Condition at phase change interface equations (5) is transformed to

(11) $$\begin{array}{c} T_{f}^{*}\left(x_{i}^{*},t^{*}\right)=1=T_{u}^{*}\left(x_{i}^{*},t^{*}\right), \end{array}$$

(12) $$\begin{array}{c} \frac{1}{K^{*}}\frac{\partial T_{f}^{*}\left(x_{i}^{*},t^{*}\right)}{\partial x^{*}}-\frac{\partial T_{u}^{*}\left(x_{i}^{*},t^{*}\right)}{\partial x^{*}}=\frac{dx_{i}^{*}}{dt^{*}}. \end{array}$$

## 3. Quasi-Steady Approximation

Due to nonlinearity of the interface energy equation, there are few exact solutions to the problems with phase change. The condition at phase change equation is nonlinear because the interface velocity *dx*_*i*_/*dt* depends on the temperature gradients. In this model, the Stefan number is taken small compared to the unity. A small Stefan number corresponds to the sensible heat which is small compared to the latent heat. The interface moves slowly for a small Stefan number, and the temperature distribution at each instant corresponds to that of steady-state. Quasi-steady approximation is justified for Ste < 0.1 [35, 36]. Setting Ste = 0 in (7) and we get

(13) $$\begin{array}{c} \frac{\partial^{2}T_{f}^{*}}{\partial x^{*2}}=0, \end{array}$$

(14) $$\begin{array}{c} \frac{\partial^{2}T_{u}^{*}}{\partial x^{*2}}+q_{m}^{*}=0. \end{array}$$

Integrating (13) and using boundary conditions given by (9) and (11), the temperature distribution in frozen region is obtained as

(15) $$\begin{array}{c} T_{f}^{*}\left(x^{*},t^{*}\right)=\frac{x^{*}}{x_{i}^{*}}. \end{array}$$

Integrating (14) with boundary conditions given by (10) and (11), the temperature distribution in the unfrozen region is given as

(16) $$\begin{array}{c} T_{u}^{*}\left(x^{*},t^{*}\right)=\frac{q_{m}^{*}}{2}\left(x_{i}^{*2}-x^{*2}\right)+q_{m}^{*}\left(x^{*}-x_{i}^{*}\right)+1. \end{array}$$

Substituting the temperature of frozen and unfrozen regions given by (15) and (16) into the condition at phase change interface given by (12), we obtain

(17) $$\begin{array}{c} \frac{q_{m}^{*}K^{*}x_{i}^{*2}-q_{m}^{*}K^{*}x_{i}^{*}+1}{K^{*}x_{i}^{*}}=\frac{dx_{i}^{*}}{dt^{*}}. \end{array}$$

Integrating (17) and utilizing the initial condition at *x*_i_*,

(18) $$\begin{array}{c} \overset{t^{*}}{\underset{0}{\int }}dt^{*}=\overset{x_{i}^{*}}{\underset{0}{\int }}\frac{K^{*}x_{i}^{*}dx_{i}^{*}}{q_{m}^{*}K^{*}x_{i}^{*2}-q_{m}^{*}K^{*}x_{i}^{*}+1}, \end{array}$$

(19) t∗=12qm∗[log⁡|qm∗K∗xi∗2−qm∗K∗xi∗+1|     +qm∗K∗∫0xi∗dxi∗qm∗K∗xi∗2−qm∗K∗xi∗+1].

In (19), the value of *q*_m_* is unknown. Due to the unknown value of *q*_m_*, there arise three possibilities. Therefore, we evaluate the above integral considering the three cases which are mentioned below.

Case 1 (*q*_m_*K* > 4, t* ≥ 0)

From (19) we have

(20) t∗=12qm∗log⁡|qm∗K∗xi∗2−qm∗K∗xi∗+1| +K∗2∫0xi∗dxi∗qm∗K∗xi∗2−qm∗K∗xi∗+1=12qm∗log⁡|qm∗K∗xi∗2−qm∗K∗xi∗+1| +12qm∗∫0xi∗dxi∗xi∗2−xi∗+(1/qm∗K∗)=12qm∗log⁡|qm∗K∗xi∗2−qm∗K∗xi∗+1|+12qm∗ ×∫0xi∗(dxi∗({xi∗−12(1+1−4qm∗K∗)}         ×{xi∗−12(1−1−4qm∗K∗)})−1)=12qm∗log⁡|qm∗K∗xi∗2−qm∗K∗xi∗+1| +K∗qm∗2K∗2−4qm∗K∗ ×log⁡|2qm∗K∗xi∗−qm∗K∗−qm∗2K∗2−4qm∗K∗2qm∗K∗xi∗−qm∗K∗+qm∗2K∗2−4qm∗K∗| −K∗qm∗2K∗2−4qm∗K∗ ×log⁡|−qm∗K∗−qm∗2K∗2−4qm∗K∗−qm∗K∗+qm∗2K∗2−4qm∗K∗|.

Case 2 (*q*_m_*K* < 4, T* ≥ 0)

From (19) we have

(21) t∗=12qm∗log⁡|qm∗K∗xi∗2−qm∗K∗xi∗+1| +K∗2∫0xi∗dxi∗qm∗K∗xi∗2−qm∗K∗xi∗+1=12qm∗log⁡|qm∗K∗xi∗2−qm∗K∗xi∗+1| +12qm∗∫0xi∗dxi∗xi∗2−xi∗+(1/qm∗K∗)=12qm∗log⁡|qm∗K∗xi∗2−qm∗K∗xi∗+1| +12qm∗∫0xi∗dxi∗(xi∗−(1/2))2+((1/qm∗K∗)−(1/4))2=12qm∗log⁡|qm∗K∗xi∗2−qm∗K∗xi∗+1| +K∗4qm∗K∗−qm∗2K∗2 ×tan−1(2qm∗K∗xi∗−qm∗K∗4qm∗K∗−qm∗2K∗2) −K∗4qm∗K∗−qm∗2K∗2 ×tan−1(−qm∗K∗4qm∗K∗−qm∗2K∗2).

Case 3 (*q*_m_*K* = 4, t* ≥ 0)

From (19) we have

(22) t∗=12qm∗log⁡|qm∗K∗xi∗2−qm∗K∗xi∗+1| +K∗2∫0xi∗dxi∗qm∗K∗xi∗2−qm∗K∗xi∗+1=12qm∗log⁡|qm∗K∗xi∗2−qm∗K∗xi∗+1| +12qm∗∫0xi∗dxi∗xi∗2−xi∗+(1/qm∗K∗)=12qm∗log⁡|qm∗K∗xi∗2−qm∗K∗xi∗+1| −12qm∗xi∗−qm∗−1qm∗.

## 4. Results and Discussion

**Figure 2:**
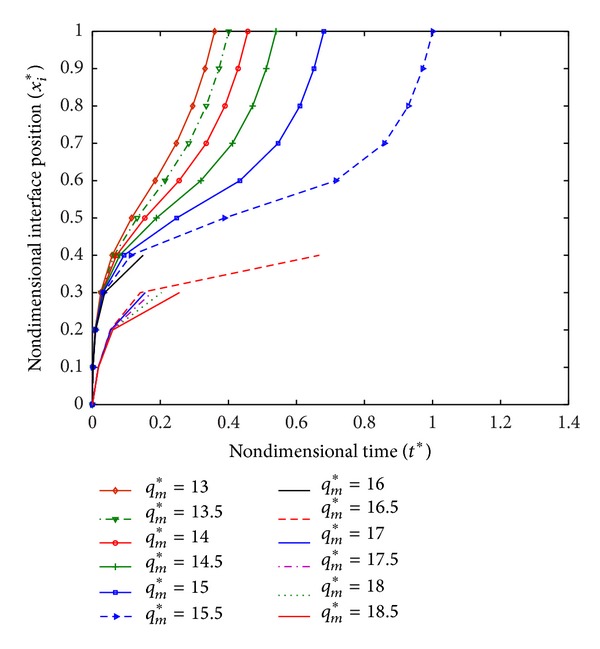
Interface position with time.

The values of parameters used are given in Table 1 [13, 23]. The position of freezing interface with time for different values of *q*_m_* is plotted in Figure 2. It is observed that when *q*_m_* < 16 (i.e., *q*_*m*_ < 940000 W/m^3^), the freezing interface reaches to the boundary *x* = *L* and the time require for solidification of the complete tissue increases with the increase in *q*_*m*_. When *q*_m_* ≥ 16 (i.e., *q*_*m*_ ≥ 940000 W/m^3^), the interface does not reach to the boundary *x* = *L*; this is because the equilibrium between cooling and heat generation is obtained before the fully freezing of tissue, and, hence, freezing interface does not move forward. Total penetration distance of freezing interface and time taken as given in Table 2 show that freezing slows down with the increase in metabolic heat generation. 

**Figure 3:**
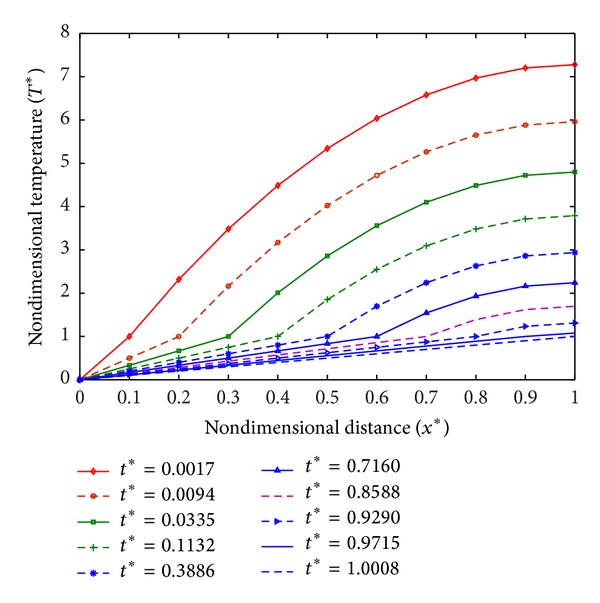
Temperature distribution at *q*_m_* = 15.5.

**Figure 4:**
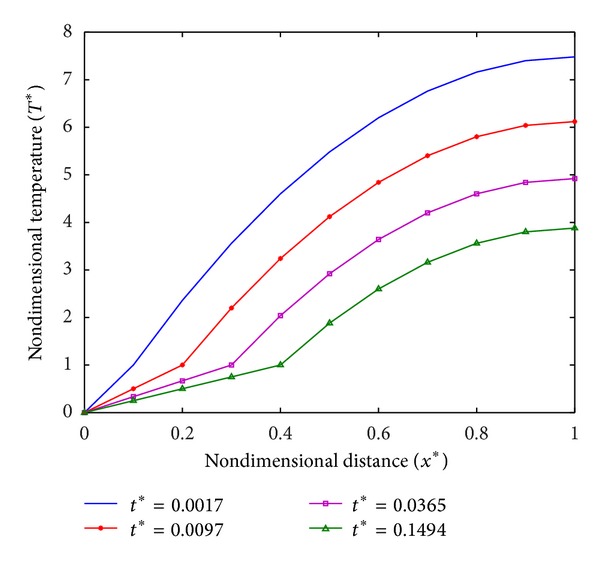
Temperature distribution at *q*_m_* = 16.

**Figure 5:**
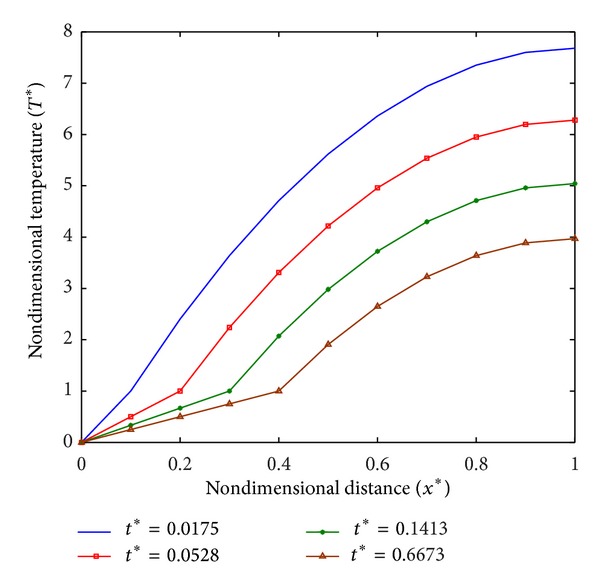
Temperature distribution at *q*_m_* = 16.5.

The temperature profiles are required to optimize the damage to diseased tissues. Temperature profiles for different values of *q*_m_*, that is, *q*_m_* = 15.5, *q*_m_* = 16, and *q*_m_* = 16.5, are plotted in Figures 3, 4, and 5, respectively. From Figure 3, it is observed that temperature in tissue decreases with the increase in time, and at t* = 1.0008 it is in frozen state. While in case of *q*_m_* = 16 as shown in Figure 4, a steady state is obtained at t* = 0.1494 and tissue is partially frozen. Similarly, for *q*_m_* ≥ 16, the partially frozen state of tissue is observed in steady state at t* = 0.6673 (Figure 5). 

## 5. Conclusion

A quasi-steady approximation is used to get the temperature profile and position of freezing interface in the biological tissue during the freezing of biological tissues for different values of metabolic heat generation. It is observed that the freezing process slows down with increase in metabolic heat generation. Freezing of the entire tissue is even not possible when the value of metabolic heat generation is extended to a higher value. This shows that metabolism has a significant effect on the freezing of biological tissues during cryosurgery. The obtained information can be used to optimize the treatment planning.

## Figures and Tables

**Table 1:** Thermal properties of tissues [13, 23].

Parameter	Value
Density of unfrozen tissue (kg/m^3^)	1050
Density of frozen tissue (kg/m^3^)	921
Specific heat of unfrozen tissue (J/kg °C)	3770
Specific heat of frozen tissue (J/kg °C)	1800
Thermal conductivity of unfrozen tissue (W/m °C)	0.5
Thermal conductivity of frozen tissue (W/m °C)	2
Latent heat (KJ/kg)	250
The phase change temperature (°C)	−8
Arterial blood temperature (°C)	37
Length of tissue (m)	0.04

**Table 2:** Penetration distance of interface and time taken for different values of *q*_m_*.

*q*_m_*	*q*_*m*_ (W/m^3^)	Interface penetration distance *x*_i_*	Time t*
13	763750	1	0.3596
13.5	793125	1	0.4009
14	822500	1	0.4571
14.5	851875	1	0.5402
15	881250	1	0.6807
15.5	910625	1	1.0008
16	940000	0.4	0.1494
16.5	989375	0.4	0.6673
17	998750	0.3	0.1554
17.5	1028125	0.3	0.1747
18	1057500	0.3	0.2036
18.5	1086875	0.3	0.2561

## Nomenclature

*x*: Distance (0 ≤ *x* ≤ *L*) (m)
*L*: Length of tissue (m)
*x*_*i*_: Position of freezing interface (m)
*t*: Time (s)
*T*: Temperature (°C)
*ρ*: Density (kg/m^3^)
*c*: Specific heat (J/kg °C)
*α*: Thermal diffusivity (m^2^/sec)
*k*: Thermal conductivity (W/m °C)
*l*: Latent heat (KJ/kg)
*q*_*m*_: Metabolic heat generation (W/m^3^)
*q*_*b*_: Blood perfusion term (W/m^3 °^C).

### Subscripts

ph: Phase change
*f*: Frozen state
*u*: Unfrozen state
*I*: Initial state (*t* = 0)
*o*: Cryoprobe.

### Superscript

∗: Dimensionless.
